# Association between inflammatory bowel disease and Parkinson’s disease: A Mendelian randomization study

**DOI:** 10.1038/s41531-022-00318-7

**Published:** 2022-05-09

**Authors:** D. Freuer, C. Meisinger

**Affiliations:** Chair of Epidemiology, University of Augsburg, University Hospital of Augsburg, Augsburg, Germany

**Keywords:** Risk factors, Gastrointestinal diseases

## Abstract

Emerging evidence from observational studies suggests an increased risk of Parkinson’s disease (PD) in patients with inflammatory bowel disease (IBD). However, to date it is not clear whether a causal relationship exists. To investigate whether IBD is causally related to PD, a two-sample Mendelian randomization study was carried out. Independent genetic instruments from the largest available genome-wide association study (GWAS) for IBD (7045 cases, 456,327 controls) including European participants were used to investigate the association with PD (56,306 cases; 1.4 million controls). The results were validated by using a second IBD sample (12,882 cases; 21,770 controls) including the main subtypes ulcerative colitis (UC; 6968 cases; 20,464 controls) and Crohn’s disease (CD; 5956 cases; 14,927 controls). The radial inverse-variance weighted (IVW) approach was used in the primary analysis, and the robustness of the findings were confirmed in a number of sensitivity analyses. Finally, the recently proposed CAUSE approach was performed. There was no evidence of an association between IBD and PD (OR_IVW_ = 0.98; 95% CI: [0.93; 1.04]; *P* = 0.48). This finding could be validated using a second sample of IBD cases (OR_IVW_ = 0.98; 95% CI: [0.95; 1.02]; *P* = 0.36). Furthermore, MR analyses did not support a causal effect of CD (OR_IVW_ = 1.00; 95% CI: [0.98; 1.03]; *P* = 0.96) or UC (OR_IVW_ = 1.02; 95% CI: [0.98; 1.06]; *P* = 0.45) on PD. The present study suggests that neither IBD nor its subtypes CD and UC causally affect Parkinson’s disease in the European population. Further research is necessary to investigate whether intestinal inflammation impacts the development of PD.

## Introduction

Parkinson’s disease (PD) is the second most common neurodegenerative disorder^1^ with 1% of the population over the age of 60 suffering from the disease^2^. Additionally, it is estimated that its prevalence will double over the next 30 years^1^. Pathophysiologically, PD is caused by a profound loss of dopaminergic neurons in the substantia nigra pars compacta, accompanied by a reduction of dopamine in the brain. A further pathophysiological hallmark of PD are filamentous protein inclusions, termed Lewiy bodies, in the surviving neurons^3,4^. Symptoms associated with the disease include cardinal motor features of bradykinesia, rigidity, resting tremor, and postural instability as well as non-motor features such as depression, cognitive decline, sleep disturbances, and obstipation^2^. Furthermore, there is evidence that chronic low-level intestinal inflammation may play a role in the development of PD by disrupting the intestinal and blood-brain barriers^5–7^. Inflammatory bowel disease (IBD), including its major subtypes ulcerative colitis (UC) and Crohn’s disease (CD), is another important disease that is characterized by chronic intestinal inflammation. As a result, the potential interplay between the inflammatory processes in IBD and PD has become a focus of interest for scientists^8^. Furthermore, a genome-wide association study (GWAS) suggested a common shared risk of genetic variants between IBD and PD^9^. Several observational studies investigating the association between IBD and PD have reported positive relationships^6,7,10,11^, while one study showed no association^12^, and two further studies found an inverse relationship^13,14^. A very recent systematic review and meta-analysis suggested an association between IBD and PD, especially for CD and UC^8^.

Since the previous study results are contradictory, a clear appraisal of the causality of an association between IBD and PD will contribute to the current evidence.

A Mendelian randomization (MR) approach tests for a causative association between an exposure and an outcome by using genetic variants, which are randomly present from conception according to Mendel’s laws of inheritance and thus independent of factors biasing observational studies. In the present study, a two-sample summary data MR analysis was performed to investigate a causal relationship between IBD (including its both conditions CD and UC) and PD.

## Results

### Genetic instruments

In the analysis investigating the causal impact of IBD on PD, 27 single nucleotide polymorphisms (SNPs) were selected as potential genetic instruments. Sixty-two, 52, and 37 SNPs were used in the validation analyses as instruments for IBD, CD, and UC, respectively. There was an overlap of two SNPs from both IBD datasets. Under the recently proposed CAUSE approach, 2688 SNPs could be used in the initial analysis with IBD and 2125; 2111; and 2136 SNPs in the validation analyses for IBD, CD, and UC, respectively.

### Main analyses

**Fig. 1:**
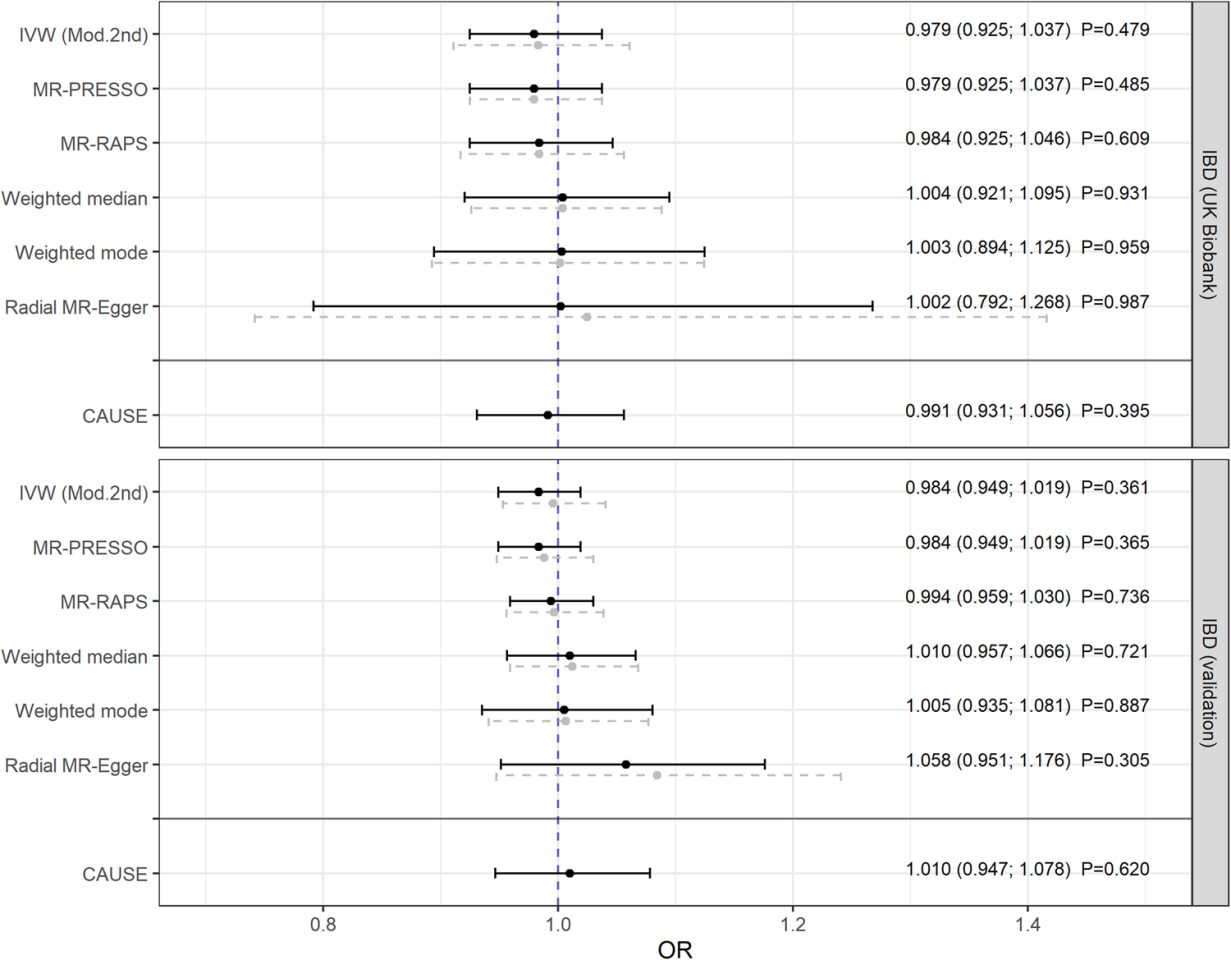
Mendelian randomization estimates given as odds ratios (ORs) and 95% confidence intervals for the causal effect of genetically predicted inflammatory bowel disease (IBD) on Parkinson’s disease (PD). The ORs can be interpreted as the average change in the outcome per 2.72-fold increase in the prevalence of the exposure. Analyses based on two different genome-wide association studies. Dashed gray lines represent the results before and black lines the results after outlier removal.

**Fig. 2:**
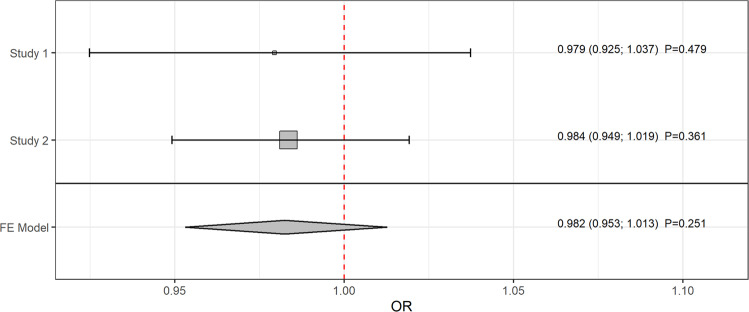
Combined causal radial IVW estimator with second-order weights from a fixed effect meta-analysis for the causal effect of genetically predicted inflammatory bowel disease on Parkinson’s disease. The shown estimates are given as odds ratios (ORs) and 95% confidence intervals and can be interpreted as the average change in the outcome per 2.72-fold increase in the prevalence of the exposure.

**Fig. 3:**
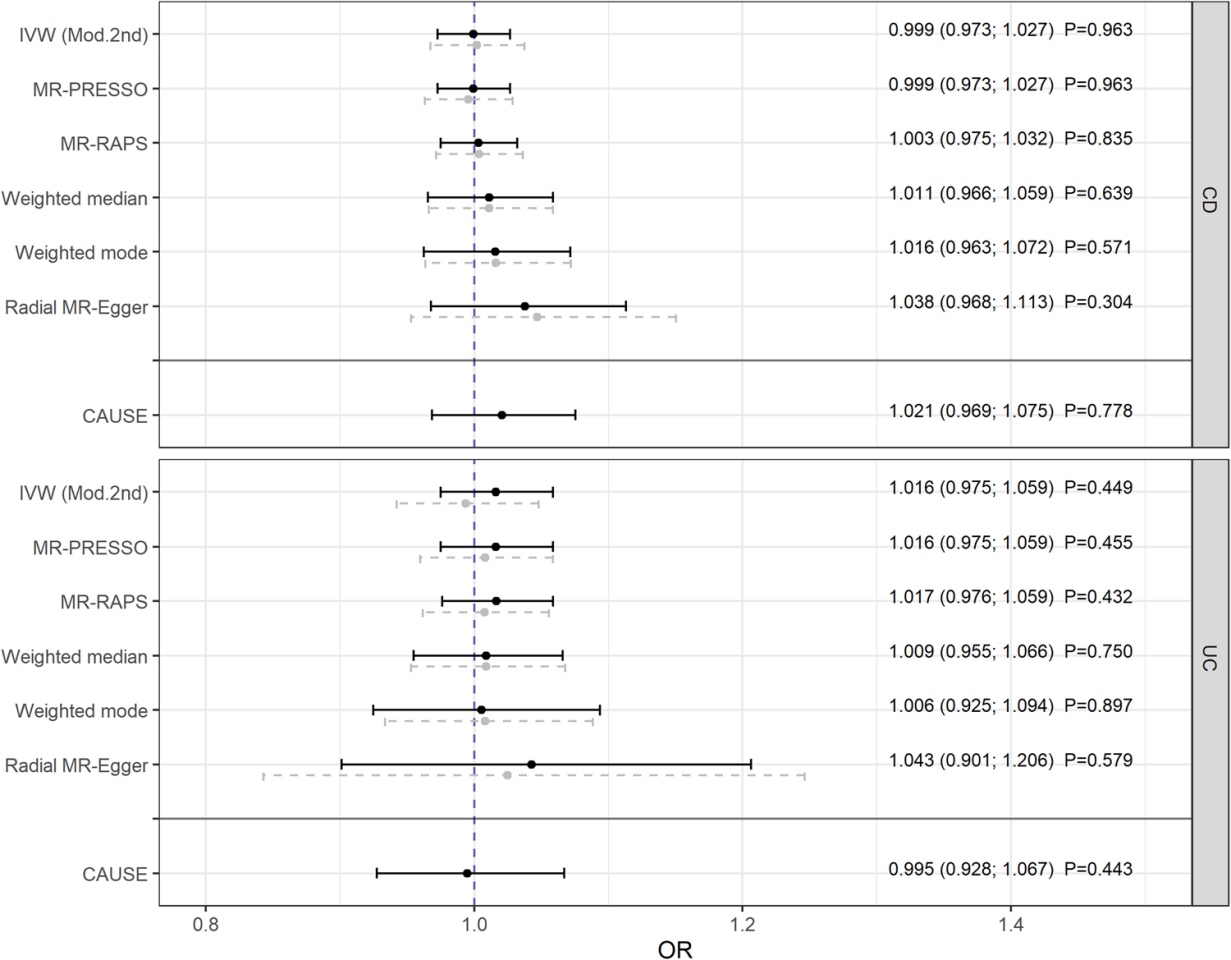
Mendelian randomization estimates given as odds ratios (ORs) and 95% confidence intervals for the causal effect of genetically predicted Crohn’s disease (CD) and ulcerative colitis (UC) on Parkinson’s disease (PD). The ORs can be interpreted as the average change in the outcome per 2.72-fold increase in the prevalence of the exposure. Dashed gray lines represent the results before and black lines the results after outlier removal.

The main analysis was conducted using the radial IVW method with modified second-order weights (obtained estimates are reported in the following). After removing pleiotropic SNPs (listed in Supplementary Table 1), there was no evidence that genetically predicted IBD was associated with PD (odds ratio (OR) = 0.979, 95% confidence interval (CI): [0.925; 1.037]; *P* = 0.479) [Fig. 1]. In the validation analysis, this result could be confirmed showing no impact of IBD as a whole on PD (OR = 0.984, 95% CI: [0.949; 1.019]; *P* = 0.361) [Fig. 1]. Furthermore, using a meta-analysis fixed effect model, the combined estimate supported the results above (OR = 0.982, 95% CI: [0.953; 1.013]; *P* = 0.251) [Fig. 2]. Additionally, there was no evidence for a causal relation of the two main subtypes of IBD, CD, and UC, to PD [Fig. 3].

Finally, despite the shared risk of genetic variants, no causal associations could be found between the three disorders and PD even within the CAUSE approach (regarding the causal estimates with 0.991 ≤ OR ≤ 1.021 [Figs. 1 and 3] or when testing the causal models against the respective model without a causal effect with 0.84 ≤ *P* ≤ 1 [Supplementary Figs. 1 and 2]).

### Sensitivity analyses and instrument strength

Neither directional pleiotropy nor between-SNP-heterogeneity could be observed regarding the tests and statistics shown in the Supplementary Table 2. Pleiotropy-robust approaches within sensitivity analyses revealed estimates supporting the results from the main analyses throughout [Figs. 1 and 3]. SNPs selected as genetic instruments explained 0.29% of the phenotypic variance regarding IBD in subjects in the UK Biobank cohort. However, in validation analyses, the explained variance by SNPs was 11.3%, 15.2%, and 8.4% in IBD, CD, and UC, respectively. The SNP-specific F-statistics, which ranged between 30 and 297 and were therefore clearly above the suggested threshold of 10, indicated the absence of weak instrument bias [Supplementary Tables 3–6].

## Discussion

In the present study, we found no evidence to support a causal role of IBD, including its main sub entities CD and UC, in the risk of PD. The findings could be confirmed in validation, meta-, and a number of sensitivity analyses with different methods.

Some prior large-scale observational studies suggested that patients with IBD may have a higher risk of developing PD^6,7,10,11^. A nationwide Danish cohort study ranging from 1977–2014 and including 76,477 patients with IBD and 7,548,259 control subjects from the general population showed a significant association between IBD and the later development of PD^7^. A significant relationship between these two diseases was also found in a nationwide population-based cohort study from Korea^10^, in a Taiwanese retrospective cohort^6^, and in a case-control^14^ and retrospective cohort study^11^ from the US. A recent systematic review and meta-analysis based on nine observational studies including 12,177,520 patients also reported a significantly higher risk of PD in patients with IBD (adjusted risk ratio = 1.24; 95% CI 1.15–1.34)^8^. In that meta-analysis, a significant relationship to PD was also reported for both CD (HR 1.33; 95% CI 1.21–1.45) and UC (HR 1.25; 95% CI 1.13–1.38)^8^. However, other studies found no significant impact of IBD on the risk of PD^15,16^. A very recent retrospective cohort study using the Truven Health Marketscan database including 154,051 IBD cases and 154,051 household age-matched controls found no statistically significant relationship between IBD and PD^16^. In a Nationwide Swedish Cohort Study based on 39,652 incident IBD patients between 2002 and 2014 and 396,520 matched control cases, 103 cases of incident PD occurred after the index date^17^. In that investigation, IBD was associated with PD^17^, but after controlling for the number of study participant medical visits, the association disappeared^17^, suggesting that the association might be explained by surveillance bias. So far, it was unclear whether there is indeed a causal relationship between IBD and PD. The present study suggests no causal relationship between genetically-predicted IBD and PD.

Our findings are in contrast to observational, experimental, and interventional studies that support an association between IBD and PD. Observational studies are susceptible to unmeasured confounders, and therefore the previously observed associations between the complex and multifactorial traits IBD and PD might be biased. Furthermore, PD may not be directly caused by the diagnosis of IBD but rather by a related inflammatory mediator that is linked to PD risk. This assumption is supported by the fact that the IBD-associated PD risk identified in the study by Peter et al. was greatly reduced among IBD patients treated with anti-inflammatory anti-TNF therapy^11^. Apart from that, the possibility that it is not necessarily the diagnosis of IBD but rather an associated inflammatory mediator that is associated with the risk of PD could not be ruled out with our MR study. A prior study showed that both rare and common variations at the leucine-rich repeat kinase 2 (*LRRK2*) gene can influence PD susceptibility^18,19^. However, rare coding variants are not captured by GWAS studies. Therefore, results could be different if different genetic instruments are used in MR analysis.

One strength of this study is that we assessed the causal effects of IBD as a whole and the two main subentities UC and CD on the risk of PD using a 2-sample MR approach. In comparison with observational research, this approach is less susceptible to confounding, reverse causation, and exposures non-differentially measured with error^20^. We applied an iterative approach, which was conservative, confirmed the consistency of estimates before and after outlier removal, and thus strengthened the evidence presented in this study. Moreover, we were able to validate our finding for the association between genetically predicted IBD and PD with a second, largely independent GWAS, resulting in similar causal estimates. Furthermore, we conducted a number of sensitivity analyses to ensure the consistency of causal estimates and to confirm the robustness of the present findings. Finally, the CAUSE approach used a much higher number of genetic variants and accounted for possible sample overlaps of exposure and outcome datasets. In summary, this analysis combines two different studies with two different main statistical approaches, a series of sensitivity analyses, and a meta-analysis estimate, which all produced similar results.

However, there are also limitations that need to be considered. There is evidence that genetic factors contribute only to a small fraction of the pathogenesis of IBD and PD diseases^21^. Moreover, the chosen genetic instruments explained 0.29% of the variance in the IBD-sample from the UK Biobank and 11.3% in the validation sample. Although a small fraction of the explained variance is typical in MR studies assessing complex traits, this issue lowers the statistical power (i.e., to reveal any relationship between IBD and PD if one exists)^22^. Finally, since the present findings are based on subjects of European descendent, generalizability to other ethnicities is limited.

The present study provides no evidence that genetically predicted IBD and also its main subtypes CD and UC are causally related to PD. Further research is necessary to examine the factors that causally impact the development of PD.

## Methods

### Study population

For IBD, summary statistics from a GWAS of the UK Biobank including European men and women (7045 mainly self-reported cases; 456,327 controls) were used^23^. For the validation analysis, summary statistics from a GWAS including European participants providing data on IBD as a whole (12,882 clinically diagnosed cases; 21,770 controls) and also on the disease entities CD (5956 clinically diagnosed cases; 14,927 controls) and UC (6968 clinically diagnosed cases; 20,464 controls) were available^24^. For the outcome PD, summary statistics from a GWAS-meta-analysis utilizing 15 datasets of Europeans (excluding the 23andMe and PDWBS (Web-Based Study of Parkinson’s Disease) studies) were used^25^. The 56,306 PD-cases consist of 37,688 sufferers and 18,618 UK Biobank proxy-cases (i.e., parental cases) and were compared to 1.4 million controls. In all original studies, ethical approval and consent to participate had been obtained. Detailed information on the cohorts has been reported elsewhere^23–25^.

### Study design

To investigate and test for a causal impact of a modifiable risk factor on an outcome, a summary-level MR analysis uses genetic variants in an instrumental variable setting. Valid instruments are characterized by the fact that they affect the outcome only through the exposure. Otherwise, when the genetic variants affect the outcome either directly or through a confounder of the exposure-outcome association, horizontal pleiotropy occurs. The SNP-specific causal effect can be estimated by the ratio of the SNP-outcome-association and SNP-exposure-association (Wald ratio). If the ratio of a valid instrument is (independent of a significant association with the outcome) different from zero, then the specific SNP indicates a cause-effect chain from the exposure to the outcome.

### Instrument selection

As possible genetic instruments, we selected SNPs with an imputation score ≥ 0.8 and associated with the respective exposure at the genome-wide significance threshold of *P* = 5 × 10^−8^. Independency of instruments was ensured by applying the PLINK clumping algorithm, which prunes SNPs in linkage disequilibrium based on the stringent cut-off of *r*^2^ = 0.001. For instruments that were missing from the outcome dataset, proxies with an LD score > 0.8 were used.

### Statistical analyses

In the main analysis, the IVW method with second-order weights was applied iteratively. In each iteration step, outliers were identified and excluded based on the SNP-specific Cochran’s Q- or Ruecker’s Q´-statistics and a type I error *α*_Q_ = 0.01. We calculated a combined causal effect estimate for the association between IBD and PD using a fixed-effects meta-analysis. In sensitivity analyses, we additionally performed the radial MR-Egger, weighted median, weighted mode, MR-PRESSO, and MR-RAPS methods, which are robust to different patterns of horizontal pleiotropy. Directional and horizontal pleiotropy were tested applying the radial MR-Egger intercept test and MR-PRESSO global test, respectively.

Finally, we used the CAUSE approach to assess the pleiotropy structure in the presence of pleiotropy and thus to assess whether the summary-level data is consistent with a causal or shared model (i.e., whether the majority of genetic variants affect an outcome through an exposure or through unobserved confounders of the exposure-outcome association)^26^. To allow pleiotropy and increase statistical power, CAUSE incorporates all available independent SNPs (i.e., genetic variants after clumping).

Regarding the weak instrument bias, we assessed the phenotypic variance explained by the genetic instruments and calculated SNP-specific F-statistics. Causal estimates before and after outlier removal were presented as ORs with 95% CIs and can be interpreted as the average change in the outcome per 2.72-fold increase in the prevalence of the respective binary exposure. The Bonferroni-corrected *α* = 0.017 was considered as the significance threshold. All analyses were mainly performed using the packages TwoSampleMR (version: 0.5.6), RadialMR (version: 1.0), MendelianRandomization (version: 0.6.0), MRPRESSO (version: 1.0), mr.raps (version: 0.2), and cause (version: 1.2.0) of the statistical software R (version: 4.1.0). In addition, the packages dplyr (version: 1.0.8), data.table (version: 1.14.2), and ggplot2 (version: 3.3.5) were used to read and manipulate the data and to create the figures.

### Reporting summary

Further information on research design is available in the Nature Research Reporting Summary linked to this article.

## Supplementary information

Supplementary Material

Reporting Summary

## Data Availability

The summary data for the IBD GWAS (UK Biobank) is available at https://cnsgenomics.com/content/data. The summary data for the second IBD GWAS and its subentities CD and UC is provided at https://www.ibdgenetics.org/downloads.html. The summary statistics for PD is available at https://drive.google.com/file/d/1FZ9UL99LAqyWnyNBxxlx6qOUlfAnublN/view.

## Code availability

The R-code is available on request from the corresponding author.
