# Supplementary material for: Association between inflammatory bowel disease and Parkinson’s disease: A Mendelian randomization study
Source: NPJ Parkinsons Dis. 2022 May 9;8:55. doi: 10.1038/s41531-022-00318-7 (PMC9085764; doi:10.1038/s41531-022-00318-7)
Supplement: Supplementary file 1 — Supplementary Material [file 41531_2022_318_MOESM1_ESM.docx]

| **Supplementary Table 1** Identified pleiotropic SNPs of the association between genetically predicted IBD, CD, and UC and Parkinson's disease based on the significance threshold 0.01 | | | | | |  |
| --- | --- | --- | --- | --- | --- | --- |
| **Exposure** | **Outcome** | **Iteration** | **SNP** | **Q statistic** | ***P*-value** | |
| IBD | parkinson | 1 | rs1297261 | 13.611 | 0.00022 | |
| IBD | parkinson | 1 | rs6671847 | 10.848 | 0.00099 | |
| IBD (validation) | parkinson | 1 | rs112401990 | 8.834 | 0.00296 | |
| IBD (validation) | parkinson | 1 | rs140892874 | 14.825 | 0.00012 | |
| IBD (validation) | parkinson | 1 | rs1736161 | 9.743 | 0.00180 | |
| IBD (validation) | parkinson | 1 | rs9272347 | 10.355 | 0.00129 | |
| IBD (validation) | parkinson | 1 | rs9370774 | 7.406 | 0.00650 | |
| IBD (validation) | parkinson | 2 | rs9370774 | 7.003 | 0.00814 | |
| CD | parkinson | 1 | rs112401990 | 9.118 | 0.00253 | |
| CD | parkinson | 1 | rs11564236 | 13.231 | 0.00028 | |
| CD | parkinson | 1 | rs1297271 | 10.087 | 0.00149 | |
| CD | parkinson | 1 | rs147684209 | 10.164 | 0.00143 | |
| UC | parkinson | 1 | rs10182512 | 8.995 | 0.00271 | |
| UC | parkinson | 1 | rs1801274 | 16.161 | 0.00006 | |
| UC | parkinson | 1 | rs28383456 | 9.140 | 0.00250 | |
| UC | parkinson | 2 | rs12612675 | 6.746 | 0.00939 | |

| **Supplementary Table 2** Results of the MR-PRESSO global and MR-Egger-intercept tests for detecting horizontal and directional pleiotropy and between SNP-heterogeneity based on the radial Mendelian randomization framework | | | | | | | | | | | | | |
| --- | --- | --- | --- | --- | --- | --- | --- | --- | --- | --- | --- | --- | --- |
| **Exposure** | **Outcome** | **PRESSO RSSobs** | **P_PRSSobs_** | **Radial-Egger intercept** | **P_intercept_** | **Cochrans Q** | **df** | **P_Q_** | **Rueckers Q'** | **P_Q'_** | **Q-Q'** | **P_Q-Q'_** | **Q'/Q** |
| IBD | parkinson | 24.459 | 0.548 | -0.156 | 0.846 | 22.808 | 24 | 0.531 | 22.78 | 0.533 | 0.029 | 0.866 | 0.999 |
| IBD (validation) | parkinson | 67.92 | 0.178 | -0.607 | 0.161 | 65.545 | 56 | 0.179 | 63.051 | 0.241 | 2.495 | 0.114 | 0.962 |
| CD | parkinson | 46.573 | 0.529 | -0.428 | 0.259 | 44.613 | 46 | 0.530 | 43.249 | 0.588 | 1.364 | 0.243 | 0.969 |
| UC | parkinson | 40.139 | 0.186 | -0.25 | 0.719 | 37.782 | 31 | 0.187 | 37.538 | 0.194 | 0.244 | 0.621 | 0.994 |

| **Supplementary Table 3** SNPs used as instruments, their build 37 positions, their associations with IBD as exposure and PD as outcome, their explained variances, and instrument strengths | | | | | | | | | | | | | | |  |
| --- | --- | --- | --- | --- | --- | --- | --- | --- | --- | --- | --- | --- | --- | --- | --- |
|  |  |  |  |  |  |  | **IBD (Exposure)** | | | | | **PD (Outcome)** | | | |
| **SNP** | **Chr** | **Pos** | **Gene** | **EA** | **OA** | **EAF** | **Beta** | **SE** | ***P*-value** | **R²** | **F-statistic** | **Beta** | **SE** | ***P*-value** | |
| rs10188217 | 2 | 61217542 | PUS10 | T | C | 0.4834 | -0.0958 | 0.0175 | 4.30E-08 | 6.48E-05 | 30.0112 | 0.0345 | 0.0169 | 4.13E-02 | |
| rs10737482 | 1 | 20173858 | RP11-91K11.2 | T | C | 0.3861 | -0.1451 | 0.0178 | 3.40E-16 | 1.44E-04 | 66.5712 | -0.0002 | 0.0176 | 9.89E-01 | |
| rs10761659 | 10 | 64445564 | ZNF365 | A | G | 0.4599 | -0.1166 | 0.0171 | 9.70E-12 | 1.00E-04 | 46.3871 | -0.0099 | 0.0169 | 5.55E-01 | |
| rs10799837 | 1 | 20135612 | RNF186 | G | A | 0.4319 | 0.1112 | 0.0174 | 1.60E-10 | 8.83E-05 | 40.9052 | -0.0129 | 0.0233 | 5.79E-01 | |
| rs11581607 | 1 | 67707690 | IL23R | G | A | 0.9333 | 0.4110 | 0.0403 | 2.20E-24 | 2.24E-04 | 103.8538 | 0.0083 | 0.0332 | 8.02E-01 | |
| rs12720356 | 19 | 10469975 | TYK2 | A | C | 0.9026 | -0.1511 | 0.0270 | 2.30E-08 | 6.74E-05 | 31.2520 | -0.0244 | 0.0297 | 4.11E-01 | |
| rs1297261 | 21 | 16812623 | AJ006998.2 | T | C | 0.5726 | 0.1228 | 0.0174 | 1.60E-12 | 1.08E-04 | 49.9796 | 0.0610 | 0.0171 | 3.64E-04 | |
| rs13384671 | 2 | 182311594 | ITGA4 | A | G | 0.6924 | -0.1045 | 0.0182 | 9.00E-09 | 7.13E-05 | 33.0392 | 0.0227 | 0.0208 | 2.75E-01 | |
| rs148844907 | 6 | 31628397 | C6orf47 | T | A | 0.9892 | -0.7566 | 0.0566 | 9.70E-41 | 3.85E-04 | 178.6185 | 0.0439 | 0.1209 | 7.17E-01 | |
| rs17264332 | 6 | 138005515 | RP11-95M15.1 | A | G | 0.7804 | -0.1185 | 0.0199 | 2.50E-09 | 7.67E-05 | 35.5613 | 0.0350 | 0.0206 | 9.03E-02 | |
| rs2652670 | 5 | 17132022 | BASP1 | G | A | 0.8520 | 0.1452 | 0.0254 | 1.10E-08 | 7.05E-05 | 32.6559 | 0.0344 | 0.0318 | 2.79E-01 | |
| rs2836878 | 21 | 40465534 | PCP4 | G | A | 0.7333 | 0.1908 | 0.0201 | 2.10E-21 | 1.95E-04 | 90.2641 | -0.0081 | 0.0196 | 6.79E-01 | |
| rs3024505 | 1 | 206939904 | IL10 | G | A | 0.8456 | -0.1719 | 0.0222 | 8.70E-15 | 1.30E-04 | 60.1646 | 0.0101 | 0.0249 | 6.85E-01 | |
| rs35788599 | 12 | 68476749 | IFNG-AS1 | G | C | 0.6153 | -0.1158 | 0.0174 | 3.20E-11 | 9.51E-05 | 44.0604 | -0.0120 | 0.0179 | 5.05E-01 | |
| rs36051895 | 9 | 4981866 | JAK2 | G | T | 0.7136 | -0.1132 | 0.0185 | 8.50E-10 | 8.12E-05 | 37.6447 | -0.0296 | 0.0192 | 1.23E-01 | |
| rs3807306 | 7 | 128580680 | IRF5 | G | T | 0.5074 | -0.0937 | 0.0170 | 3.70E-08 | 6.53E-05 | 30.2771 | 0.0067 | 0.0172 | 6.96E-01 | |
| rs3828058 | 1 | 151786281 | RORC | G | A | 0.6292 | -0.0980 | 0.0177 | 3.20E-08 | 6.60E-05 | 30.5832 | -0.0049 | 0.0197 | 8.02E-01 | |
| rs4129133 | 10 | 101284570 | RP11-129J12.1 | T | C | 0.4850 | 0.1124 | 0.0170 | 3.40E-11 | 9.48E-05 | 43.9133 | 0.0233 | 0.0168 | 1.65E-01 | |
| rs4551125 | 5 | 40438684 | RP11-357F12.1 | G | A | 0.3931 | -0.1083 | 0.0176 | 7.50E-10 | 8.18E-05 | 37.8894 | -0.0040 | 0.0172 | 8.16E-01 | |
| rs55722650 | 5 | 131607300 | PDLIM4 | C | T | 0.5794 | -0.1003 | 0.0171 | 4.50E-09 | 7.43E-05 | 34.4111 | -0.0032 | 0.0177 | 8.58E-01 | |
| rs59998884 | 21 | 45618114 | AP001057.1 | T | C | 0.3959 | 0.1066 | 0.0175 | 1.00E-09 | 8.04E-05 | 37.2357 | 0.0090 | 0.0173 | 6.01E-01 | |
| rs6017342 | 20 | 43065028 | HNF4A | A | C | 0.4833 | -0.1308 | 0.0170 | 1.60E-14 | 1.27E-04 | 58.9370 | 0.0071 | 0.0173 | 6.83E-01 | |
| rs6671847 | 1 | 161478810 | FCGR2A | G | A | 0.4902 | 0.1211 | 0.0171 | 1.60E-12 | 1.08E-04 | 49.9442 | -0.0607 | 0.0178 | 6.58E-04 | |
| rs6961243 | 7 | 107521404 | DLD | G | A | 0.5900 | 0.1024 | 0.0175 | 5.00E-09 | 7.38E-05 | 34.1883 | -0.0165 | 0.0172 | 3.38E-01 | |
| rs72810950 | 10 | 90822640 | snoU13 | T | C | 0.9035 | 0.1744 | 0.0308 | 1.50E-08 | 6.91E-05 | 32.0213 | 0.0149 | 0.0336 | 6.58E-01 | |
| rs7554511 | 1 | 200877562 | C1orf106 | C | A | 0.7056 | 0.1332 | 0.0191 | 2.90E-12 | 1.05E-04 | 48.7278 | -0.0248 | 0.0188 | 1.86E-01 | |
| rs7936312 | 11 | 76293726 | RP11-672A2.7 | G | T | 0.5224 | -0.1109 | 0.0170 | 6.70E-11 | 9.19E-05 | 42.6071 | 0.0328 | 0.0171 | 5.51E-02 | |

| **Supplementary Table 4** SNPs used as instruments, their build 37 positions, their associations with IBD as exposure and PD as outcome, their explained variances, and instrument strengths | | | | | | | | | | | | | | |  |
| --- | --- | --- | --- | --- | --- | --- | --- | --- | --- | --- | --- | --- | --- | --- | --- |
|  |  |  |  |  |  |  | **IBD (validation) (Exposure)** | | | | | **PD (Outcome)** | | | |
| **SNP** | **Chr** | **Pos** | **Gene** | **EA** | **OA** | **EAF** | **Beta** | **SE** | ***P*-value** | **R²** | **F-statistic** | **Beta** | **SE** | ***P*-value** | |
| rs1003342 | 22 | 30570022 | HORMAD2 | A | G | 0.4850 | 0.0950 | 0.0168 | 1.67E-08 | 9.18E-04 | 31.8418 | 0.0161 | 0.0168 | 3.38E-01 | |
| rs10045431 | 5 | 158814533 | AC008697.1 | A | C | 0.2630 | -0.1774 | 0.0189 | 6.59E-21 | 2.53E-03 | 87.9830 | 0.0130 | 0.0184 | 4.79E-01 | |
| rs10175585 | 2 | 103076107 | IL18RAP | A | G | 0.6990 | -0.1332 | 0.0202 | 3.89E-11 | 1.26E-03 | 43.6679 | -0.0054 | 0.0205 | 7.94E-01 | |
| rs10737481 | 1 | 20171514 | RP11-91K11.2 | T | G | 0.4250 | -0.1411 | 0.0170 | 1.19E-16 | 1.98E-03 | 68.6274 | -0.0022 | 0.0169 | 8.96E-01 | |
| rs10761659 | 10 | 64445564 | ZNF365 | A | G | 0.4240 | -0.1619 | 0.0172 | 4.07E-21 | 2.56E-03 | 88.9338 | -0.0099 | 0.0169 | 5.55E-01 | |
| rs10800314 | 1 | 161472789 | FCGR2A | A | C | 0.6310 | -0.1431 | 0.0179 | 1.17E-15 | 1.85E-03 | 64.1107 | 0.0412 | 0.0180 | 2.18E-02 | |
| rs11209026 | 1 | 67705958 | IL23R | A | G | 0.0322 | -0.7263 | 0.0422 | 1.76E-66 | 8.49E-03 | 296.6392 | -0.0087 | 0.0332 | 7.92E-01 | |
| rs11236797 | 11 | 76299649 | RP11-672A2.7 | A | C | 0.4920 | 0.1557 | 0.0170 | 4.75E-20 | 2.42E-03 | 84.0758 | -0.0391 | 0.0172 | 2.34E-02 | |
| rs112401990 | 2 | 61199327 | PUS10 | A | G | 0.3980 | 0.1422 | 0.0174 | 2.84E-16 | 1.93E-03 | 66.9081 | -0.0532 | 0.0177 | 2.72E-03 | |
| rs112694524 | 2 | 43453721 | ZFP36L2 | A | G | 0.0976 | 0.1883 | 0.0303 | 5.39E-10 | 1.11E-03 | 38.5274 | 0.0155 | 0.0363 | 6.69E-01 | |
| rs11548656 | 16 | 81916912 | PLCG2 | A | G | 0.9710 | 0.2928 | 0.0507 | 7.72E-09 | 9.61E-04 | 33.3430 | 0.0396 | 0.0580 | 4.95E-01 | |
| rs11677953 | 2 | 219121663 | GPBAR1 | A | G | 0.4270 | 0.0976 | 0.0171 | 1.05E-08 | 9.44E-04 | 32.7390 | -0.0466 | 0.0183 | 1.09E-02 | |
| rs12446550 | 16 | 28543381 | NUPR1 | A | G | 0.4260 | 0.1078 | 0.0171 | 2.78E-10 | 1.15E-03 | 39.8190 | -0.0395 | 0.0180 | 2.80E-02 | |
| rs1250573 | 10 | 81042475 | ZMIZ1 | A | G | 0.2800 | -0.1136 | 0.0190 | 2.21E-09 | 1.03E-03 | 35.7780 | -0.0079 | 0.0247 | 7.48E-01 | |
| rs12764283 | 10 | 35530460 | CCNY | A | G | 0.3550 | 0.1266 | 0.0179 | 1.57E-12 | 1.44E-03 | 49.9523 | 0.0025 | 0.0179 | 8.90E-01 | |
| rs12936409 | 17 | 38043649 | ZPBP2 | T | C | 0.4990 | 0.1457 | 0.0168 | 3.87E-18 | 2.17E-03 | 75.3833 | 0.0200 | 0.0168 | 2.34E-01 | |
| rs131657 | 22 | 21917550 | UBE2L3 | A | T | 0.2180 | 0.1365 | 0.0212 | 1.24E-10 | 1.19E-03 | 41.4032 | 0.0170 | 0.0216 | 4.31E-01 | |
| rs13178036 | 5 | 40218529 | RP11-357F12.1 | C | G | 0.3550 | 0.1009 | 0.0184 | 4.13E-08 | 8.68E-04 | 30.0862 | 0.0027 | 0.0202 | 8.92E-01 | |
| rs140892874 | 12 | 40824798 | MUC19 | T | C | 0.9650 | -0.4096 | 0.0512 | 1.28E-15 | 1.84E-03 | 63.9343 | -0.2066 | 0.0541 | 1.33E-04 | |
| rs142770866 | 19 | 10525372 | CDC37 | A | G | 0.0899 | 0.2300 | 0.0336 | 8.14E-12 | 1.35E-03 | 46.7281 | 0.0114 | 0.0322 | 7.22E-01 | |
| rs148844907 | 6 | 31628397 | C6orf47 | A | T | 0.0159 | 1.1375 | 0.0963 | 3.63E-32 | 4.01E-03 | 139.3782 | -0.0439 | 0.1209 | 7.17E-01 | |
| rs1551399 | 8 | 126539965 | RP11-136O12.2 | A | C | 0.3710 | -0.1013 | 0.0173 | 5.01E-09 | 9.86E-04 | 34.1828 | 0.0057 | 0.0173 | 7.41E-01 | |
| rs1736161 | 21 | 16833222 | AJ006998.2 | A | G | 0.4080 | -0.1233 | 0.0174 | 1.34E-12 | 1.45E-03 | 50.2712 | -0.0538 | 0.0174 | 2.02E-03 | |
| rs17800987 | 5 | 150323428 | ZNF300P1 | A | G | 0.8970 | -0.2017 | 0.0305 | 3.71E-11 | 1.26E-03 | 43.7602 | 0.0153 | 0.0300 | 6.10E-01 | |
| rs1873625 | 3 | 49666964 | BSN | A | C | 0.3480 | 0.1773 | 0.0179 | 3.71E-23 | 2.83E-03 | 98.2339 | 0.0032 | 0.0182 | 8.59E-01 | |
| rs1887428 | 9 | 4984530 | JAK2 | C | G | 0.5980 | -0.1716 | 0.0178 | 6.65E-22 | 2.66E-03 | 92.5196 | -0.0199 | 0.0200 | 3.19E-01 | |
| rs2076756 | 16 | 50756881 | NOD2 | A | G | 0.7080 | -0.1876 | 0.0186 | 5.59E-24 | 2.93E-03 | 101.9803 | -0.0264 | 0.0193 | 1.71E-01 | |
| rs2193041 | 12 | 68502110 | IFNG-AS1 | A | G | 0.5930 | -0.1337 | 0.0172 | 6.91E-15 | 1.75E-03 | 60.6207 | -0.0138 | 0.0181 | 4.46E-01 | |
| rs2241878 | 2 | 234183718 | ATG16L1 | T | C | 0.4370 | -0.1480 | 0.0169 | 1.75E-18 | 2.22E-03 | 76.9551 | 0.0030 | 0.0168 | 8.57E-01 | |
| rs2542147 | 18 | 12775851 | RP11-973H7.1 | T | G | 0.8240 | -0.1513 | 0.0227 | 2.79E-11 | 1.28E-03 | 44.3199 | 0.0340 | 0.0233 | 1.45E-01 | |
| rs254559 | 5 | 134444982 | C5orf66 | A | C | 0.4140 | 0.1028 | 0.0172 | 2.08E-09 | 1.03E-03 | 35.8971 | -0.0001 | 0.0217 | 9.96E-01 | |
| rs2836882 | 21 | 40466570 | PCP4 | A | G | 0.2350 | -0.1963 | 0.0201 | 1.49E-22 | 2.75E-03 | 95.4797 | 0.0026 | 0.0189 | 8.91E-01 | |
| rs3024493 | 1 | 206943968 | IL10 | A | C | 0.1860 | 0.2130 | 0.0222 | 8.48E-22 | 2.65E-03 | 92.0389 | -0.0048 | 0.0239 | 8.40E-01 | |
| rs3091316 | 17 | 32593974 | CCL7 | A | G | 0.2540 | -0.1125 | 0.0191 | 3.59E-09 | 1.00E-03 | 34.8339 | 0.0250 | 0.0189 | 1.87E-01 | |
| rs34190331 | 6 | 111840820 | TRAF3IP2-AS1 | A | G | 0.0941 | 0.1769 | 0.0303 | 5.39E-09 | 9.81E-04 | 34.0399 | -0.0148 | 0.0339 | 6.64E-01 | |
| rs35260072 | 5 | 131630852 | SLC22A4 | A | C | 0.5480 | -0.1422 | 0.0170 | 7.07E-17 | 2.01E-03 | 69.6504 | 0.0034 | 0.0174 | 8.43E-01 | |
| rs35730213 | 1 | 200874229 | C1orf106 | C | G | 0.2450 | -0.1514 | 0.0194 | 6.91E-15 | 1.75E-03 | 60.6210 | 0.0227 | 0.0189 | 2.29E-01 | |
| rs3850378 | 14 | 88417517 | GALC | T | C | 0.8960 | -0.1551 | 0.0282 | 3.80E-08 | 8.72E-04 | 30.2467 | 0.0177 | 0.0297 | 5.52E-01 | |
| rs4077515 | 9 | 139266496 | CARD9 | T | C | 0.4500 | 0.1794 | 0.0172 | 1.50E-25 | 3.14E-03 | 109.1497 | 0.0301 | 0.0171 | 7.82E-02 | |
| rs4246905 | 9 | 117553249 | TNFSF15 | T | C | 0.2500 | -0.1630 | 0.0197 | 1.42E-16 | 1.97E-03 | 68.2810 | 0.0114 | 0.0188 | 5.44E-01 | |
| rs444210 | 6 | 167390242 | RP1-167A14.2 | A | G | 0.4360 | -0.1095 | 0.0168 | 7.39E-11 | 1.22E-03 | 42.4102 | 0.0390 | 0.0170 | 2.21E-02 | |
| rs4676408 | 2 | 241574401 | GPR35 | A | G | 0.5270 | 0.1181 | 0.0181 | 6.62E-11 | 1.23E-03 | 42.6260 | -0.0066 | 0.0184 | 7.20E-01 | |
| rs4712528 | 6 | 20678430 | CDKAL1 | C | G | 0.7990 | 0.1226 | 0.0207 | 3.07E-09 | 1.01E-03 | 35.1344 | 0.0085 | 0.0209 | 6.85E-01 | |
| rs4730272 | 7 | 107478227 | PIGCP2 | A | G | 0.5050 | 0.1341 | 0.0178 | 4.50E-14 | 1.64E-03 | 56.9328 | -0.0020 | 0.0175 | 9.07E-01 | |
| rs56062135 | 15 | 67455630 | SMAD3 | T | C | 0.2520 | 0.1509 | 0.0198 | 2.64E-14 | 1.67E-03 | 57.9826 | -0.0078 | 0.0199 | 6.95E-01 | |
| rs6062496 | 20 | 62329099 | TNFRSF6B | A | G | 0.6000 | 0.1650 | 0.0180 | 5.48E-20 | 2.41E-03 | 83.7935 | -0.0420 | 0.0178 | 1.82E-02 | |
| rs62126615 | 19 | 33749710 | CTD-2540B15.12 | T | C | 0.1900 | 0.1432 | 0.0231 | 5.33E-10 | 1.11E-03 | 38.5503 | -0.0022 | 0.0219 | 9.19E-01 | |
| rs6584283 | 10 | 101290301 | NKX2-3 | T | C | 0.5170 | 0.1803 | 0.0169 | 1.70E-26 | 3.26E-03 | 113.4651 | 0.0262 | 0.0169 | 1.20E-01 | |
| rs6826501 | 4 | 36076676 | ARAP2 | T | C | 0.5210 | -0.0928 | 0.0169 | 4.12E-08 | 8.68E-04 | 30.0899 | -0.0073 | 0.0177 | 6.80E-01 | |
| rs6873866 | 5 | 96247810 | ERAP2 | T | C | 0.4850 | 0.1070 | 0.0176 | 1.09E-09 | 1.07E-03 | 37.1637 | -0.0163 | 0.0171 | 3.41E-01 | |
| rs6880778 | 5 | 40399096 | RP11-357F12.1 | A | G | 0.3510 | -0.1878 | 0.0173 | 2.14E-27 | 3.38E-03 | 117.5721 | -0.0035 | 0.0173 | 8.41E-01 | |
| rs6911490 | 6 | 106522027 | ATG5 | T | C | 0.2340 | 0.1428 | 0.0208 | 6.82E-12 | 1.36E-03 | 47.0755 | 0.0165 | 0.0207 | 4.26E-01 | |
| rs6927172 | 6 | 138002175 | RP11-95M15.1 | C | G | 0.7750 | -0.1103 | 0.0202 | 4.65E-08 | 8.61E-04 | 29.8578 | 0.0354 | 0.0207 | 8.69E-02 | |
| rs72798422 | 16 | 50866917 | CTD-2034I21.1 | T | C | 0.9520 | -0.2776 | 0.0431 | 1.19E-10 | 1.20E-03 | 41.4806 | 0.0558 | 0.0644 | 3.86E-01 | |
| rs72844760 | 6 | 31463371 | MICB | A | G | 0.0690 | 0.2279 | 0.0360 | 2.43E-10 | 1.16E-03 | 40.0823 | 0.0529 | 0.0442 | 2.32E-01 | |
| rs7285952 | 22 | 39733096 | SCUBE1 | T | G | 0.8560 | 0.1760 | 0.0235 | 7.60E-14 | 1.61E-03 | 55.9028 | -0.0522 | 0.0254 | 3.98E-02 | |
| rs744166 | 17 | 40514201 | STAT3 | A | G | 0.6070 | 0.1207 | 0.0172 | 2.16E-12 | 1.42E-03 | 49.3329 | 0.0201 | 0.0177 | 2.57E-01 | |
| rs7523335 | 1 | 8180210 | RP11-431K24.1 | A | G | 0.1640 | -0.1405 | 0.0225 | 4.16E-10 | 1.13E-03 | 39.0350 | 0.0005 | 0.0245 | 9.83E-01 | |
| rs8134436 | 21 | 45616497 | AP001057.1 | C | G | 0.4180 | 0.1447 | 0.0170 | 2.02E-17 | 2.08E-03 | 72.1227 | 0.0098 | 0.0173 | 5.71E-01 | |
| rs9272347 | 6 | 32604394 | HLA-DQA1 | T | C | 0.7070 | 0.2191 | 0.0201 | 9.95E-28 | 3.43E-03 | 119.0945 | 0.0760 | 0.0239 | 1.50E-03 | |
| rs9370774 | 6 | 14721897 | RP11-146I2.1 | T | C | 0.8100 | 0.1307 | 0.0219 | 2.54E-09 | 1.02E-03 | 35.5091 | -0.0762 | 0.0278 | 6.18E-03 | |
| rs9934775 | 16 | 50383077 | BRD7 | T | C | 0.1500 | -0.1396 | 0.0232 | 1.72E-09 | 1.05E-03 | 36.2712 | -0.0108 | 0.0304 | 7.22E-01 | |

| **Supplementary Table 5** SNPs used as instruments, their build 37 positions, their associations with IBD as exposure and PD as outcome, their explained variances, and instrument strengths | | | | | | | | | | | | | | |  |
| --- | --- | --- | --- | --- | --- | --- | --- | --- | --- | --- | --- | --- | --- | --- | --- |
|  |  |  |  |  |  |  | **CD (Exposure)** | | | | | **PD (Outcome)** | | | |
| **SNP** | **Chr** | **Pos** | **Gene** | **EA** | **OA** | **EAF** | **Beta** | **SE** | ***P*-value** | **R²** | **F-statistic** | **Beta** | **SE** | ***P*-value** | |
| rs1056441 | 20 | 62370349 | LIME1 | T | C | 0.2780 | -0.1670 | 0.0255 | 5.44E-11 | 2.06E-03 | 43.0077 | 0.0241 | 0.0185 | 1.91E-01 | |
| rs10748781 | 10 | 101283330 | - | A | C | 0.5180 | -0.2191 | 0.0238 | 3.72E-20 | 4.03E-03 | 84.5584 | -0.0291 | 0.0179 | 1.04E-01 | |
| rs10761659 | 10 | 64445564 | ZNF365 | A | G | 0.4130 | -0.2120 | 0.0237 | 3.42E-19 | 3.82E-03 | 80.1712 | -0.0099 | 0.0169 | 5.55E-01 | |
| rs11209026 | 1 | 67705958 | IL23R | A | G | 0.0263 | -0.9952 | 0.0639 | 1.05E-54 | 1.15E-02 | 242.6033 | -0.0087 | 0.0332 | 7.92E-01 | |
| rs11236797 | 11 | 76299649 | RP11-672A2.7 | A | C | 0.5040 | 0.1811 | 0.0231 | 4.85E-15 | 2.93E-03 | 61.3130 | -0.0391 | 0.0172 | 2.34E-02 | |
| rs112401990 | 2 | 61199327 | PUS10 | A | G | 0.3970 | 0.1322 | 0.0237 | 2.35E-08 | 1.49E-03 | 31.1785 | -0.0532 | 0.0177 | 2.72E-03 | |
| rs11564236 | 12 | 40828306 | MUC19 | A | T | 0.9520 | -0.5191 | 0.0595 | 2.85E-18 | 3.63E-03 | 75.9869 | -0.1974 | 0.0540 | 2.55E-04 | |
| rs12194825 | 6 | 20835260 | CDKAL1 | A | T | 0.1680 | -0.1719 | 0.0298 | 8.00E-09 | 1.59E-03 | 33.2719 | -0.0004 | 0.0224 | 9.84E-01 | |
| rs1250573 | 10 | 81042475 | ZMIZ1 | A | G | 0.2610 | -0.1709 | 0.0264 | 9.01E-11 | 2.01E-03 | 42.0221 | -0.0079 | 0.0247 | 7.48E-01 | |
| rs12692254 | 2 | 234161211 | ATG16L1 | A | T | 0.4030 | -0.3014 | 0.0232 | 1.86E-38 | 7.99E-03 | 168.1509 | 0.0035 | 0.0169 | 8.34E-01 | |
| rs12717899 | 5 | 141482341 | PRELID2 | T | G | 0.8110 | 0.1592 | 0.0289 | 3.59E-08 | 1.45E-03 | 30.3541 | 0.0085 | 0.0208 | 6.82E-01 | |
| rs1297271 | 21 | 16823163 | AJ006998.2 | T | C | 0.4020 | -0.1549 | 0.0237 | 6.28E-11 | 2.04E-03 | 42.7278 | -0.0546 | 0.0171 | 1.38E-03 | |
| rs13135092 | 4 | 103198082 | SLC39A8 | A | G | 0.8910 | -0.2215 | 0.0389 | 1.21E-08 | 1.55E-03 | 32.4676 | 0.0301 | 0.0315 | 3.40E-01 | |
| rs1456896 | 7 | 50304461 | AC020743.3 | T | C | 0.7190 | 0.1393 | 0.0251 | 2.90E-08 | 1.47E-03 | 30.7724 | -0.0260 | 0.0181 | 1.52E-01 | |
| rs147018773 | 5 | 150238521 | IRGM | T | C | 0.1200 | 0.3217 | 0.0375 | 8.89E-18 | 3.52E-03 | 73.7384 | -0.0077 | 0.0285 | 7.88E-01 | |
| rs147684209 | 16 | 28867061 | SH2B1 | T | C | 0.6120 | -0.1549 | 0.0244 | 2.34E-10 | 1.92E-03 | 40.1594 | 0.0555 | 0.0175 | 1.49E-03 | |
| rs148844907 | 6 | 31628397 | C6orf47 | A | T | 0.0125 | 0.9580 | 0.1419 | 1.47E-11 | 2.18E-03 | 45.5680 | -0.0439 | 0.1209 | 7.17E-01 | |
| rs151314883 | 22 | 39735087 | SCUBE1 | A | G | 0.1390 | -0.2240 | 0.0327 | 7.12E-12 | 2.25E-03 | 46.9885 | 0.0530 | 0.0255 | 3.78E-02 | |
| rs1873625 | 3 | 49666964 | BSN | A | C | 0.3510 | 0.1807 | 0.0243 | 1.09E-13 | 2.64E-03 | 55.1851 | 0.0032 | 0.0182 | 8.59E-01 | |
| rs1887428 | 9 | 4984530 | JAK2 | C | G | 0.5990 | -0.1681 | 0.0243 | 4.22E-12 | 2.29E-03 | 48.0130 | -0.0199 | 0.0200 | 3.19E-01 | |
| rs1932990 | 13 | 44460242 | LACC1 | T | C | 0.2760 | 0.1529 | 0.0263 | 6.02E-09 | 1.62E-03 | 33.8246 | -0.0171 | 0.0199 | 3.92E-01 | |
| rs2076756 | 16 | 50756881 | NOD2 | A | G | 0.6570 | -0.3998 | 0.0242 | 3.25E-61 | 1.29E-02 | 272.4686 | -0.0264 | 0.0193 | 1.71E-01 | |
| rs2129944 | 19 | 10516198 | CDC37 | T | G | 0.7280 | 0.1562 | 0.0271 | 7.81E-09 | 1.59E-03 | 33.3187 | 0.0072 | 0.0352 | 8.38E-01 | |
| rs2188962 | 5 | 131770805 | C5orf56 | T | C | 0.4760 | 0.2124 | 0.0228 | 1.36E-20 | 4.13E-03 | 86.5453 | -0.0061 | 0.0170 | 7.22E-01 | |
| rs2505640 | 10 | 35459497 | CREM | A | G | 0.3800 | 0.1457 | 0.0237 | 7.61E-10 | 1.81E-03 | 37.8535 | 0.0002 | 0.0181 | 9.91E-01 | |
| rs281379 | 19 | 49214274 | MAMSTR | A | G | 0.5050 | 0.1398 | 0.0238 | 4.26E-09 | 1.65E-03 | 34.4960 | -0.0021 | 0.0171 | 9.01E-01 | |
| rs28701841 | 6 | 106530330 | ATG5 | A | G | 0.1280 | 0.2243 | 0.0373 | 1.85E-09 | 1.73E-03 | 36.1211 | 0.0172 | 0.0296 | 5.61E-01 | |
| rs3024505 | 1 | 206939904 | IL10 | A | G | 0.1780 | 0.1779 | 0.0302 | 3.91E-09 | 1.66E-03 | 34.6672 | -0.0101 | 0.0249 | 6.85E-01 | |
| rs3091315 | 17 | 32593665 | CCL7 | A | G | 0.7580 | 0.1795 | 0.0263 | 9.52E-12 | 2.22E-03 | 46.4202 | -0.0266 | 0.0189 | 1.58E-01 | |
| rs3810936 | 9 | 117552885 | TNFSF15 | T | C | 0.2730 | -0.2078 | 0.0263 | 2.46E-15 | 2.99E-03 | 62.6496 | 0.0080 | 0.0182 | 6.60E-01 | |
| rs4077515 | 9 | 139266496 | CARD9 | T | C | 0.4520 | 0.2159 | 0.0235 | 4.37E-20 | 4.02E-03 | 84.2374 | 0.0301 | 0.0171 | 7.82E-02 | |
| rs41315816 | 6 | 31944851 | STK19 | T | C | 0.9120 | -0.3046 | 0.0437 | 3.23E-12 | 2.32E-03 | 48.5379 | -0.0622 | 0.0400 | 1.19E-01 | |
| rs444210 | 6 | 167390242 | RP1-167A14.2 | A | G | 0.4260 | -0.1634 | 0.0229 | 1.03E-12 | 2.43E-03 | 50.7908 | 0.0390 | 0.0170 | 2.21E-02 | |
| rs4820091 | 22 | 21940189 | UBE2L3 | T | G | 0.7810 | -0.1717 | 0.0282 | 1.22E-09 | 1.77E-03 | 36.9384 | -0.0147 | 0.0218 | 5.00E-01 | |
| rs4851586 | 2 | 103064264 | IL18RAP | T | C | 0.2610 | 0.1689 | 0.0261 | 9.94E-11 | 2.00E-03 | 41.8302 | 0.0051 | 0.0204 | 8.04E-01 | |
| rs4902642 | 14 | 69210199 | RNU6-921P | A | G | 0.3870 | -0.1292 | 0.0236 | 4.34E-08 | 1.43E-03 | 29.9888 | -0.0190 | 0.0171 | 2.68E-01 | |
| rs56062135 | 15 | 67455630 | SMAD3 | T | C | 0.2550 | 0.1931 | 0.0269 | 7.45E-13 | 2.46E-03 | 51.4163 | -0.0078 | 0.0199 | 6.95E-01 | |
| rs6588243 | 1 | 67603383 | C1orf141 | A | C | 0.3850 | -0.1317 | 0.0234 | 1.78E-08 | 1.52E-03 | 31.7211 | 0.0148 | 0.0172 | 3.88E-01 | |
| rs6704109 | 1 | 172857050 | RP1-15D23.2 | T | C | 0.2840 | 0.2020 | 0.0256 | 2.77E-15 | 2.98E-03 | 62.4175 | 0.0215 | 0.0196 | 2.72E-01 | |
| rs6873866 | 5 | 96247810 | ERAP2 | T | C | 0.4880 | 0.1681 | 0.0239 | 2.07E-12 | 2.36E-03 | 49.4165 | -0.0163 | 0.0171 | 3.41E-01 | |
| rs697693 | 1 | 7886424 | PER3 | A | G | 0.2200 | 0.1723 | 0.0281 | 8.36E-10 | 1.80E-03 | 37.6718 | 0.0061 | 0.0230 | 7.91E-01 | |
| rs7276302 | 21 | 45614159 | - | A | G | 0.4210 | 0.1716 | 0.0231 | 1.23E-13 | 2.63E-03 | 54.9613 | 0.0099 | 0.0173 | 5.67E-01 | |
| rs72798422 | 16 | 50866917 | CTD-2034I21.1 | T | C | 0.9330 | -0.5904 | 0.0508 | 3.19E-31 | 6.43E-03 | 135.0535 | 0.0558 | 0.0644 | 3.86E-01 | |
| rs744166 | 17 | 40514201 | STAT3 | A | G | 0.6140 | 0.1293 | 0.0233 | 2.92E-08 | 1.47E-03 | 30.7550 | 0.0201 | 0.0177 | 2.57E-01 | |
| rs7543234 | 1 | 155253308 | HCN3 | T | C | 0.2590 | 0.1555 | 0.0267 | 6.10E-09 | 1.62E-03 | 33.7999 | -0.0227 | 0.0195 | 2.46E-01 | |
| rs7713270 | 5 | 40440063 | RP11-357F12.1 | T | C | 0.6710 | 0.2966 | 0.0241 | 6.97E-35 | 7.22E-03 | 151.7969 | 0.0044 | 0.0172 | 8.00E-01 | |
| rs7714401 | 5 | 158852944 | AC008697.1 | A | T | 0.3560 | 0.1594 | 0.0244 | 6.20E-11 | 2.04E-03 | 42.7513 | 0.0032 | 0.0194 | 8.68E-01 | |
| rs78487399 | 2 | 43809347 | THADA | C | G | 0.1150 | 0.2259 | 0.0370 | 1.03E-09 | 1.78E-03 | 37.2618 | 0.0043 | 0.0298 | 8.87E-01 | |
| rs80262450 | 18 | 12818922 | PTPN2 | A | G | 0.1320 | 0.2831 | 0.0353 | 1.08E-15 | 3.07E-03 | 64.2690 | -0.0165 | 0.0290 | 5.70E-01 | |
| rs8178977 | 19 | 1106477 | GPX4 | C | G | 0.2610 | 0.1928 | 0.0274 | 2.06E-12 | 2.36E-03 | 49.4251 | 0.0088 | 0.0254 | 7.30E-01 | |
| rs907092 | 17 | 37922259 | IKZF3 | A | G | 0.4950 | 0.1304 | 0.0228 | 1.01E-08 | 1.57E-03 | 32.8168 | 0.0107 | 0.0169 | 5.28E-01 | |
| rs921720 | 8 | 126534671 | RP11-136O12.2 | A | G | 0.3590 | -0.1629 | 0.0237 | 6.40E-12 | 2.26E-03 | 47.1998 | 0.0066 | 0.0174 | 7.03E-01 | |

| **Supplementary Table 6** SNPs used as instruments, their build 37 positions, their associations with IBD as exposure and PD as outcome, their explained variances, and instrument strengths | | | | | | | | | | | | | | |  |
| --- | --- | --- | --- | --- | --- | --- | --- | --- | --- | --- | --- | --- | --- | --- | --- |
|  |  |  |  |  |  |  | **UC (Exposure)** | | | | | **PD (Outcome)** | | | |
| **SNP** | **Chr** | **Pos** | **Gene** | **EA** | **OA** | **EAF** | **Beta** | **SE** | ***P*-value** | **R²** | **F-statistic** | **Beta** | **SE** | ***P*-value** | |
| rs10182512 | 2 | 61189469 | PUS10 | A | G | 0.3760 | 0.1608 | 0.0223 | 5.19E-13 | 1.90E-03 | 52.1264 | -0.0538 | 0.0176 | 2.26E-03 | |
| rs10272963 | 7 | 107486902 | PIGCP2 | T | C | 0.3940 | -0.1719 | 0.0216 | 1.69E-15 | 2.31E-03 | 63.3909 | 0.0184 | 0.0169 | 2.79E-01 | |
| rs10737481 | 1 | 20171514 | RP11-91K11.2 | T | G | 0.4040 | -0.2501 | 0.0216 | 4.37E-31 | 4.88E-03 | 134.4341 | -0.0022 | 0.0169 | 8.96E-01 | |
| rs10917545 | 1 | 20128177 | TMCO4 | A | G | 0.8680 | -0.1851 | 0.0335 | 3.29E-08 | 1.11E-03 | 30.5239 | 0.0055 | 0.0374 | 8.83E-01 | |
| rs11209026 | 1 | 67705958 | IL23R | A | G | 0.0370 | -0.5617 | 0.0517 | 1.58E-27 | 4.29E-03 | 118.1757 | -0.0087 | 0.0332 | 7.92E-01 | |
| rs114152040 | 5 | 40444986 | RP11-357F12.1 | A | G | 0.0397 | 0.3396 | 0.0623 | 4.95E-08 | 1.08E-03 | 29.7345 | 0.0456 | 0.0504 | 3.66E-01 | |
| rs12067391 | 1 | 8163858 | RP11-431K24.1 | T | G | 0.8330 | 0.1634 | 0.0285 | 9.67E-09 | 1.20E-03 | 32.9041 | -0.0004 | 0.0242 | 9.86E-01 | |
| rs12612675 | 2 | 219133137 | AAMP | A | G | 0.5830 | -0.1229 | 0.0219 | 1.98E-08 | 1.15E-03 | 31.5147 | 0.0461 | 0.0182 | 1.14E-02 | |
| rs12817473 | 12 | 68497408 | IFNG-AS1 | A | G | 0.5850 | -0.1907 | 0.0217 | 1.71E-18 | 2.80E-03 | 76.9951 | -0.0125 | 0.0176 | 4.79E-01 | |
| rs1359946 | 13 | 27536972 | RP11-545M8.4 | A | G | 0.2140 | 0.1583 | 0.0269 | 3.84E-09 | 1.26E-03 | 34.7017 | -0.0060 | 0.0221 | 7.87E-01 | |
| rs137845 | 22 | 50439430 | IL17REL | A | G | 0.4620 | -0.1182 | 0.0212 | 2.38E-08 | 1.13E-03 | 31.1578 | -0.0080 | 0.0171 | 6.39E-01 | |
| rs148844907 | 6 | 31628397 | C6orf47 | A | T | 0.0187 | 1.3413 | 0.1089 | 7.17E-35 | 5.50E-03 | 151.7422 | -0.0439 | 0.1209 | 7.17E-01 | |
| rs1801274 | 1 | 161479745 | FCGR2A | A | G | 0.5460 | 0.1829 | 0.0217 | 3.78E-17 | 2.58E-03 | 70.8843 | -0.0703 | 0.0172 | 4.38E-05 | |
| rs1886731 | 1 | 2472081 | RP3-395M20.12 | T | C | 0.5440 | 0.1405 | 0.0222 | 2.25E-10 | 1.46E-03 | 40.2301 | 0.0359 | 0.0179 | 4.50E-02 | |
| rs1887428 | 9 | 4984530 | JAK2 | C | G | 0.5960 | -0.1767 | 0.0224 | 3.36E-15 | 2.26E-03 | 62.0416 | -0.0199 | 0.0200 | 3.19E-01 | |
| rs2212434 | 11 | 76281593 | EMSY | T | C | 0.4790 | 0.1419 | 0.0213 | 2.46E-11 | 1.62E-03 | 44.5598 | -0.0360 | 0.0173 | 3.75E-02 | |
| rs254559 | 5 | 134444982 | C5orf66 | A | C | 0.4250 | 0.1243 | 0.0215 | 7.63E-09 | 1.21E-03 | 33.3653 | -0.0001 | 0.0217 | 9.96E-01 | |
| rs28383456 | 6 | 32609453 | HLA-DQA1 | T | C | 0.2890 | -0.3373 | 0.0256 | 1.07E-39 | 6.30E-03 | 173.8270 | -0.1182 | 0.0398 | 2.94E-03 | |
| rs3024493 | 1 | 206943968 | IL10 | A | C | 0.1920 | 0.2363 | 0.0276 | 1.09E-17 | 2.67E-03 | 73.3335 | -0.0048 | 0.0239 | 8.40E-01 | |
| rs35730213 | 1 | 200874229 | C1orf106 | C | G | 0.2450 | -0.1670 | 0.0245 | 8.82E-12 | 1.69E-03 | 46.5722 | 0.0227 | 0.0189 | 2.29E-01 | |
| rs3829111 | 9 | 139269483 | CARD9 | A | G | 0.4480 | 0.1563 | 0.0214 | 2.89E-13 | 1.94E-03 | 53.2750 | 0.0315 | 0.0171 | 6.51E-02 | |
| rs4574921 | 9 | 117538334 | RP11-428F18.2 | T | C | 0.7580 | 0.1506 | 0.0256 | 4.24E-09 | 1.26E-03 | 34.5083 | -0.0190 | 0.0202 | 3.47E-01 | |
| rs4676410 | 2 | 241563739 | GPR35 | A | G | 0.2210 | 0.2078 | 0.0284 | 2.46E-13 | 1.95E-03 | 53.5950 | 0.0127 | 0.0222 | 5.67E-01 | |
| rs483905 | 11 | 96023427 | MAML2 | A | G | 0.3100 | 0.1289 | 0.0228 | 1.57E-08 | 1.16E-03 | 31.9599 | -0.0122 | 0.0220 | 5.79E-01 | |
| rs484356 | 11 | 114406639 | NXPE1 | C | G | 0.6910 | 0.1342 | 0.0228 | 3.95E-09 | 1.26E-03 | 34.6457 | 0.0088 | 0.0177 | 6.20E-01 | |
| rs56167332 | 5 | 158827769 | AC008697.1 | A | C | 0.3640 | 0.1516 | 0.0231 | 5.30E-11 | 1.57E-03 | 43.0604 | -0.0042 | 0.0197 | 8.30E-01 | |
| rs6062496 | 20 | 62329099 | TNFRSF6B | A | G | 0.5980 | 0.1585 | 0.0224 | 1.47E-12 | 1.82E-03 | 50.0871 | -0.0420 | 0.0178 | 1.82E-02 | |
| rs6933404 | 6 | 137959235 | AL356739.1 | T | C | 0.7620 | -0.1668 | 0.0252 | 3.69E-11 | 1.59E-03 | 43.7712 | 0.0292 | 0.0210 | 1.65E-01 | |
| rs7282490 | 21 | 45615741 | - | A | G | 0.5790 | -0.1397 | 0.0214 | 7.08E-11 | 1.55E-03 | 42.4948 | -0.0080 | 0.0172 | 6.41E-01 | |
| rs7752873 | 6 | 106579332 | ATG5 | T | C | 0.1480 | 0.1823 | 0.0303 | 1.83E-09 | 1.32E-03 | 36.1410 | -0.0014 | 0.0261 | 9.59E-01 | |
| rs7911680 | 10 | 101293468 | NKX2-3 | A | C | 0.5390 | 0.1718 | 0.0213 | 8.27E-16 | 2.36E-03 | 64.8007 | 0.0280 | 0.0168 | 9.61E-02 | |
| rs798502 | 7 | 2789880 | GNA12 | A | C | 0.7370 | 0.1365 | 0.0240 | 1.21E-08 | 1.18E-03 | 32.4636 | 0.0509 | 0.0229 | 2.62E-02 | |
| rs9272514 | 6 | 32606385 | HLA-DQA1 | T | C | 0.2450 | -0.4016 | 0.0267 | 4.00E-51 | 8.18E-03 | 226.1923 | -0.0572 | 0.0287 | 4.59E-02 | |
| rs9823546 | 3 | 49705512 | BSN | A | T | 0.3410 | 0.1769 | 0.0223 | 2.29E-15 | 2.28E-03 | 62.7971 | 0.0016 | 0.0182 | 9.31E-01 | |
| rs9891174 | 17 | 38031802 | ZPBP2 | A | T | 0.4980 | 0.1452 | 0.0212 | 7.17E-12 | 1.71E-03 | 46.9781 | 0.0198 | 0.0168 | 2.40E-01 | |
| rs989960 | 7 | 107445727 | SLC26A3 | T | C | 0.4070 | -0.1291 | 0.0215 | 1.77E-09 | 1.32E-03 | 36.2135 | -0.0002 | 0.0171 | 9.92E-01 | |
| rs9977672 | 21 | 40463283 | PCP4 | A | G | 0.2170 | -0.2450 | 0.0261 | 6.21E-21 | 3.20E-03 | 88.0989 | 0.0014 | 0.0193 | 9.40E-01 | |

| **IBD - Discovery** | | | | | | | | | | | |
| --- | --- | --- | --- | --- | --- | --- | --- | --- | --- | --- | --- |
| 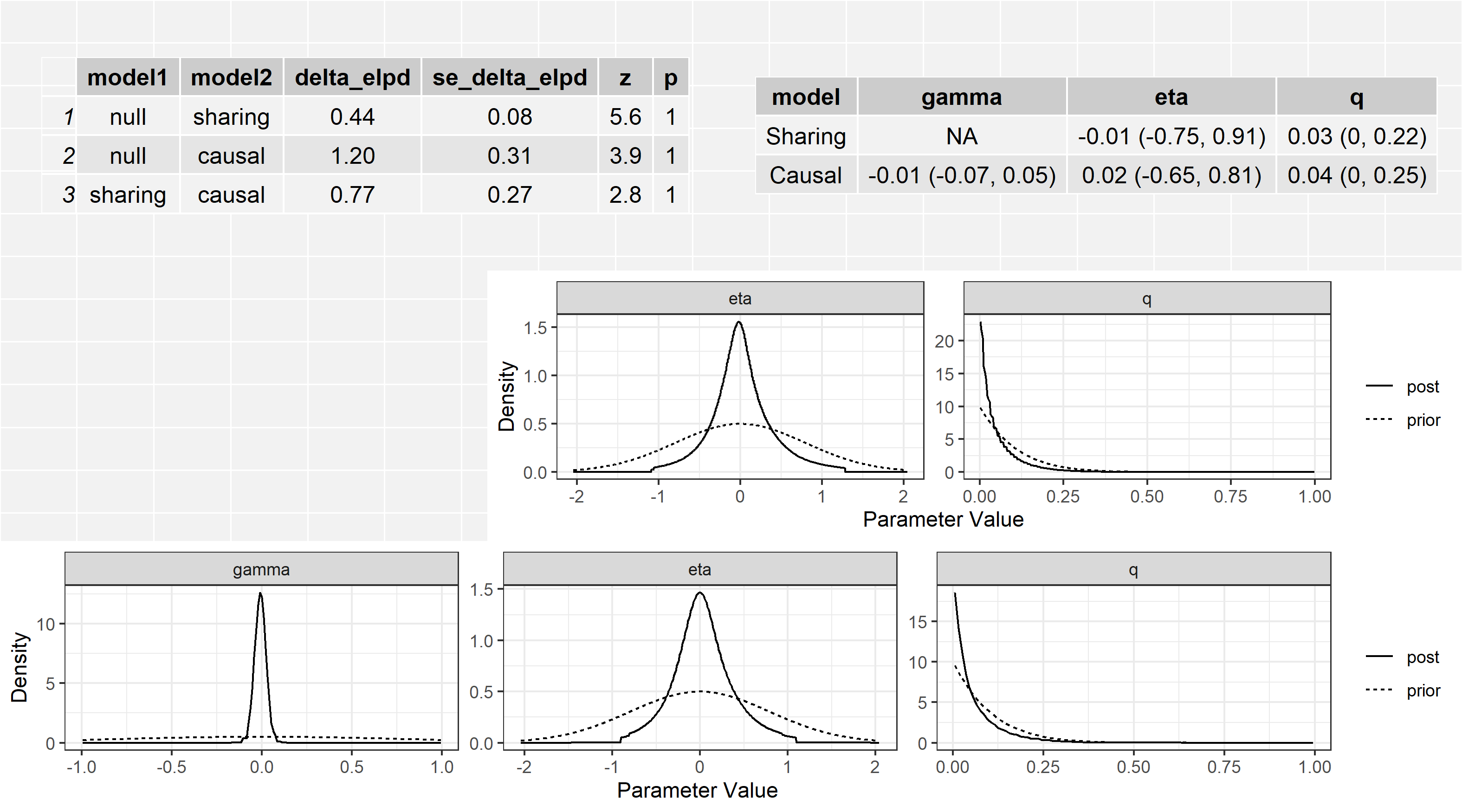   \|  \| \| --- \| |  |  |  |  |  |  |  |  |  |  |  |
|  |  |  |  |  |  |  |  |  |  |  |  |
|  |  |  |  |  |  |  |  |  |  |  |  |
|  |  |  |  |  |  |  |  |  |  |  |  |
|  |  |  |  |  |  |  |  |  |  |  |  |
|  |  |  |  |  |  |  |  |  |  |  |  |
|  |  |  |  |  |  |  |  |  |  |  |  |
|  |  |  |  |  |  |  |  |  |  |  |  |
|  |  |  |  |  |  |  |  |  |  |  |  |
|  |  |  |  |  |  |  |  |  |  |  |  |
|  |  |  |  |  |  |  |  |  |  |  |  |
|  |  |  |  |  |  |  |  |  |  |  |  |
|  |  |  |  |  |  |  |  |  |  |  |  |
|  |  |  |  |  |  |  |  |  |  |  |  |
|  |  |  |  |  |  |  |  |  |  |  |  |
|  |  |  |  |  |  |  |  |  |  |  |  |
|  |  |  |  |  |  |  |  |  |  |  |  |
|  |  |  |  |  |  |  |  |  |  |  |  |
|  |  |  |  |  |  |  |  |  |  |  |  |
|  |  |  |  |  |  |  |  |  |  |  |  |
|  |  |  |  |  |  |  |  |  |  |  |  |
|  |  |  |  |  |  |  |  |  |  |  |  |
|  |  |  |  |  |  |  |  |  |  |  |  |
|  |  |  |  |  |  |  |  |  |  |  |  |
|  |  |  |  |  |  |  |  |  |  |  |  |
|  |  |  |  |  |  |  |  |  |  |  |  |
|  |  |  |  |  |  |  |  |  |  |  |  |
|  |  |  |  |  |  |  |  |  |  |  |  |
| **IBD - Validation** | | | | | | | | | | | |
| 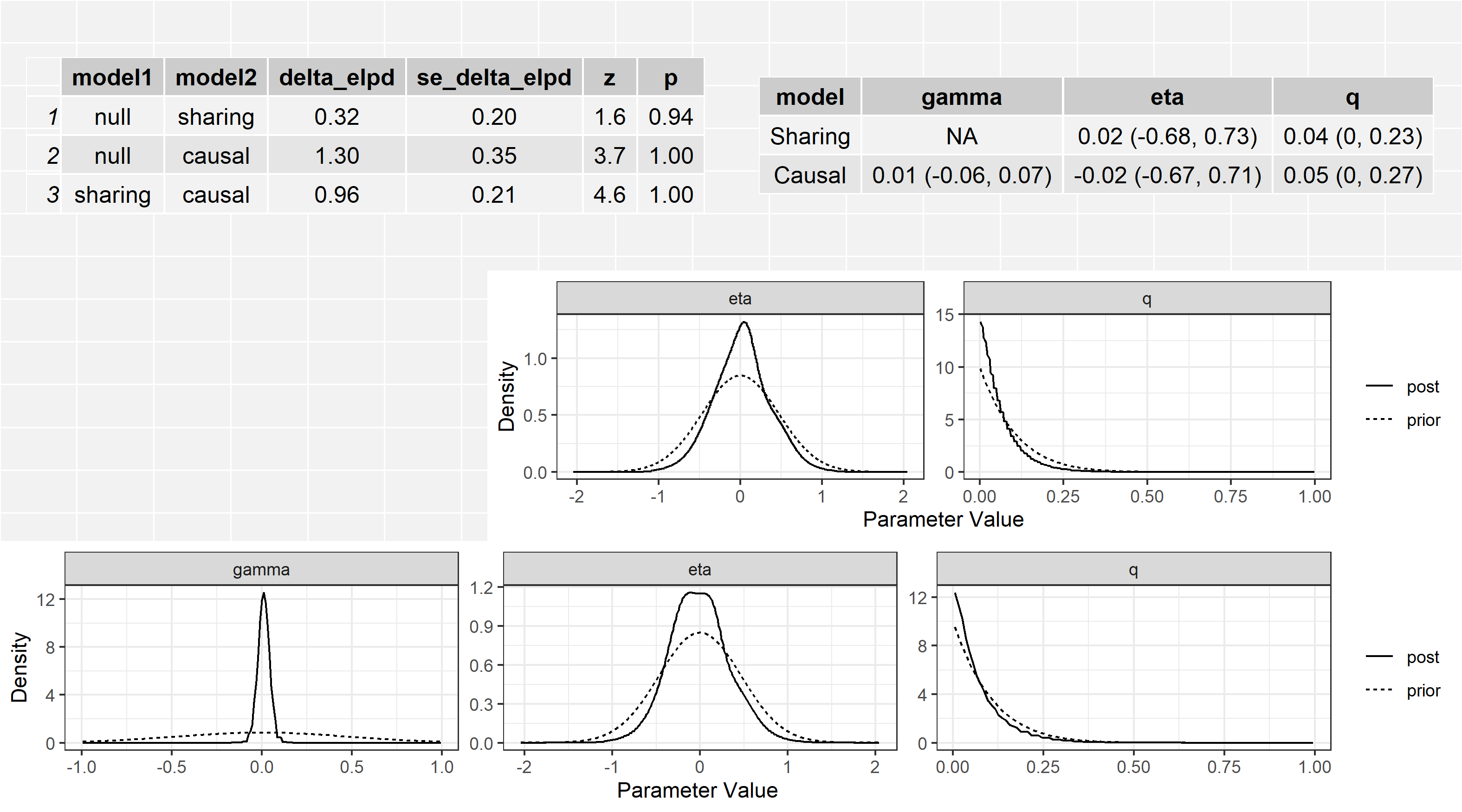   \|  \| \| --- \| |  |  |  |  |  |  |  |  |  |  |  |
|  |  |  |  |  |  |  |  |  |  |  |  |
|  |  |  |  |  |  |  |  |  |  |  |  |
|  |  |  |  |  |  |  |  |  |  |  |  |
|  |  |  |  |  |  |  |  |  |  |  |  |
|  |  |  |  |  |  |  |  |  |  |  |  |
|  |  |  |  |  |  |  |  |  |  |  |  |
|  |  |  |  |  |  |  |  |  |  |  |  |
|  |  |  |  |  |  |  |  |  |  |  |  |
|  |  |  |  |  |  |  |  |  |  |  |  |
|  |  |  |  |  |  |  |  |  |  |  |  |
|  |  |  |  |  |  |  |  |  |  |  |  |
|  |  |  |  |  |  |  |  |  |  |  |  |
|  |  |  |  |  |  |  |  |  |  |  |  |
|  |  |  |  |  |  |  |  |  |  |  |  |
|  |  |  |  |  |  |  |  |  |  |  |  |
|  |  |  |  |  |  |  |  |  |  |  |  |
|  |  |  |  |  |  |  |  |  |  |  |  |
|  |  |  |  |  |  |  |  |  |  |  |  |
|  |  |  |  |  |  |  |  |  |  |  |  |
|  |  |  |  |  |  |  |  |  |  |  |  |
|  |  |  |  |  |  |  |  |  |  |  |  |
|  |  |  |  |  |  |  |  |  |  |  |  |
|  |  |  |  |  |  |  |  |  |  |  |  |
|  |  |  |  |  |  |  |  |  |  |  |  |
|  |  |  |  |  |  |  |  |  |  |  |  |
| **Supplementary Figure 1** Estimates and model comparison from the CAUSE approach investigating the causal effect of IBD on Parkinson's disease based on two independent GWAS for IBD | | | | | | | | | | | |
| \| **CD** \| \| \| \| \| \| \| \| \| \| \| \| \| --- \| --- \| --- \| --- \| --- \| --- \| --- \| --- \| --- \| --- \| --- \| --- \| \| 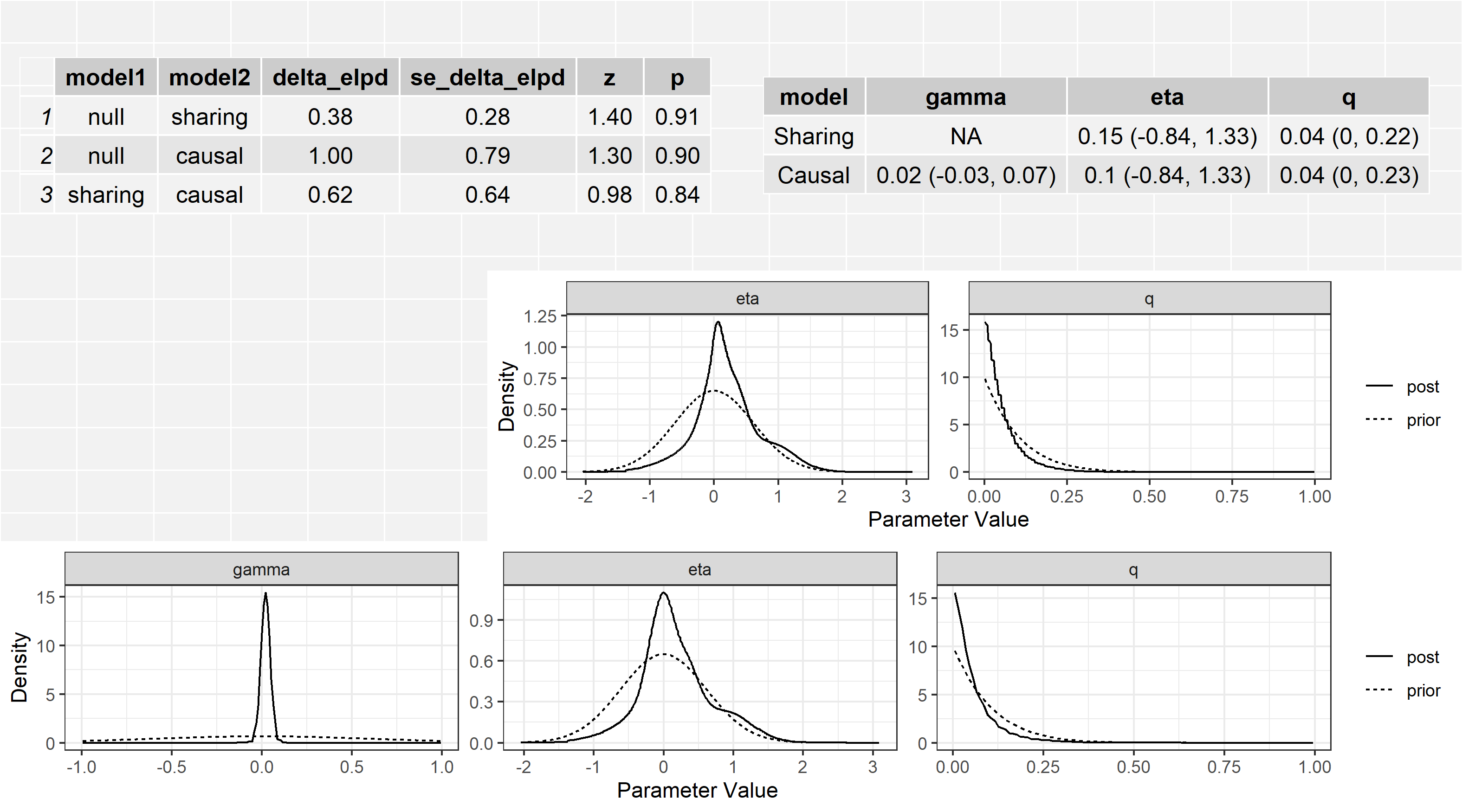   \|  \| \| --- \| \|  \|  \|  \|  \|  \|  \|  \|  \|  \|  \|  \| \|  \|  \|  \|  \|  \|  \|  \|  \|  \|  \|  \|  \| \|  \|  \|  \|  \|  \|  \|  \|  \|  \|  \|  \|  \| \|  \|  \|  \|  \|  \|  \|  \|  \|  \|  \|  \|  \| \|  \|  \|  \|  \|  \|  \|  \|  \|  \|  \|  \|  \| \|  \|  \|  \|  \|  \|  \|  \|  \|  \|  \|  \|  \| \|  \|  \|  \|  \|  \|  \|  \|  \|  \|  \|  \|  \| \|  \|  \|  \|  \|  \|  \|  \|  \|  \|  \|  \|  \| \|  \|  \|  \|  \|  \|  \|  \|  \|  \|  \|  \|  \| \|  \|  \|  \|  \|  \|  \|  \|  \|  \|  \|  \|  \| \|  \|  \|  \|  \|  \|  \|  \|  \|  \|  \|  \|  \| \|  \|  \|  \|  \|  \|  \|  \|  \|  \|  \|  \|  \| \|  \|  \|  \|  \|  \|  \|  \|  \|  \|  \|  \|  \| \|  \|  \|  \|  \|  \|  \|  \|  \|  \|  \|  \|  \| \|  \|  \|  \|  \|  \|  \|  \|  \|  \|  \|  \|  \| \|  \|  \|  \|  \|  \|  \|  \|  \|  \|  \|  \|  \| \|  \|  \|  \|  \|  \|  \|  \|  \|  \|  \|  \|  \| \|  \|  \|  \|  \|  \|  \|  \|  \|  \|  \|  \|  \| \|  \|  \|  \|  \|  \|  \|  \|  \|  \|  \|  \|  \| \|  \|  \|  \|  \|  \|  \|  \|  \|  \|  \|  \|  \| \|  \|  \|  \|  \|  \|  \|  \|  \|  \|  \|  \|  \| \|  \|  \|  \|  \|  \|  \|  \|  \|  \|  \|  \|  \| \|  \|  \|  \|  \|  \|  \|  \|  \|  \|  \|  \|  \| \|  \|  \|  \|  \|  \|  \|  \|  \|  \|  \|  \|  \| \|  \|  \|  \|  \|  \|  \|  \|  \|  \|  \|  \|  \| \|  \|  \|  \|  \|  \|  \|  \|  \|  \|  \|  \|  \| \|  \|  \|  \|  \|  \|  \|  \|  \|  \|  \|  \|  \| \|  \|  \|  \|  \|  \|  \|  \|  \|  \|  \|  \|  \| \| **UC** \| \| \| \| \| \| \| \| \| \| \| \| \| 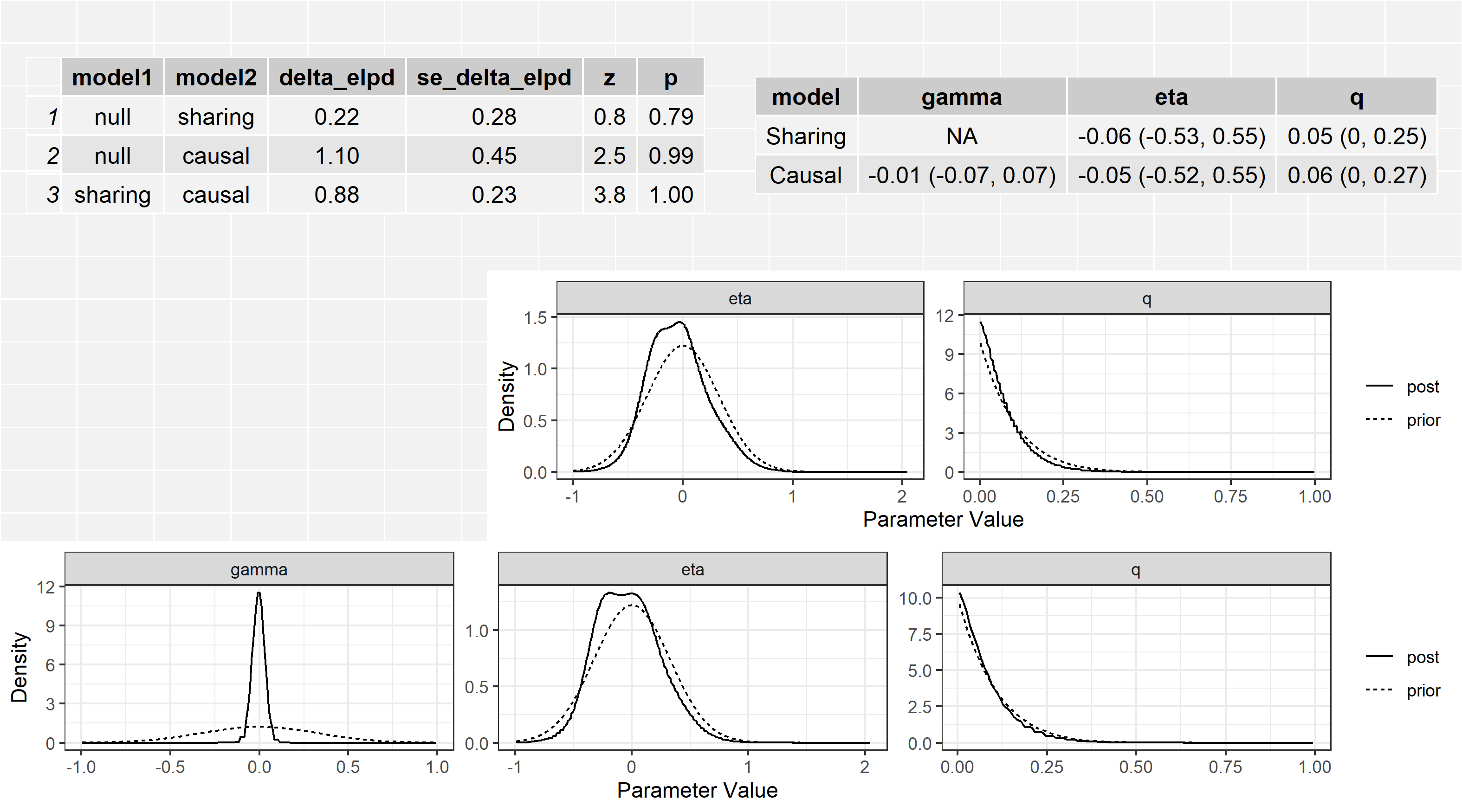   \|  \| \| --- \| \|  \|  \|  \|  \|  \|  \|  \|  \|  \|  \|  \| \|  \|  \|  \|  \|  \|  \|  \|  \|  \|  \|  \|  \| \|  \|  \|  \|  \|  \|  \|  \|  \|  \|  \|  \|  \| \|  \|  \|  \|  \|  \|  \|  \|  \|  \|  \|  \|  \| \|  \|  \|  \|  \|  \|  \|  \|  \|  \|  \|  \|  \| \|  \|  \|  \|  \|  \|  \|  \|  \|  \|  \|  \|  \| \|  \|  \|  \|  \|  \|  \|  \|  \|  \|  \|  \|  \| \|  \|  \|  \|  \|  \|  \|  \|  \|  \|  \|  \|  \| \|  \|  \|  \|  \|  \|  \|  \|  \|  \|  \|  \|  \| \|  \|  \|  \|  \|  \|  \|  \|  \|  \|  \|  \|  \| \|  \|  \|  \|  \|  \|  \|  \|  \|  \|  \|  \|  \| \|  \|  \|  \|  \|  \|  \|  \|  \|  \|  \|  \|  \| \|  \|  \|  \|  \|  \|  \|  \|  \|  \|  \|  \|  \| \|  \|  \|  \|  \|  \|  \|  \|  \|  \|  \|  \|  \| \|  \|  \|  \|  \|  \|  \|  \|  \|  \|  \|  \|  \| \|  \|  \|  \|  \|  \|  \|  \|  \|  \|  \|  \|  \| \|  \|  \|  \|  \|  \|  \|  \|  \|  \|  \|  \|  \| \|  \|  \|  \|  \|  \|  \|  \|  \|  \|  \|  \|  \| \|  \|  \|  \|  \|  \|  \|  \|  \|  \|  \|  \|  \| \|  \|  \|  \|  \|  \|  \|  \|  \|  \|  \|  \|  \| \|  \|  \|  \|  \|  \|  \|  \|  \|  \|  \|  \|  \| \|  \|  \|  \|  \|  \|  \|  \|  \|  \|  \|  \|  \| \|  \|  \|  \|  \|  \|  \|  \|  \|  \|  \|  \|  \| \|  \|  \|  \|  \|  \|  \|  \|  \|  \|  \|  \|  \| \|  \|  \|  \|  \|  \|  \|  \|  \|  \|  \|  \|  \| \|  \|  \|  \|  \|  \|  \|  \|  \|  \|  \|  \|  \| \|  \|  \|  \|  \|  \|  \|  \|  \|  \|  \|  \|  \| | | | | | | | | | | | |
| **Supplementary Figure 2** Estimates and model comparison from the CAUSE approach investigating the causal effect of both CD and UC on Parkinson's disease | | | | | | | | | | | |
